# Transformation by a nucleotide-activated P2Y receptor is mediated by activation of Gα_i_, Gα_q _and Rho-dependent signaling pathways

**DOI:** 10.1186/1750-2187-5-11

**Published:** 2010-07-23

**Authors:** Anurag Singh, José L Boyer, Channing J Der, Irene E Zohn

**Affiliations:** 1 Linebergher Comprehensive Cancer Center, University of North Carolina at Chapel Hill, NC 27599, USA; 2 Department of Pharmacology, Chapel Hill, NC 27599, USA; 3 Center for Neuroscience Research, Children's Research Institute, Children's National Medical Center, Washington, DC 20010, USA; 4 Department of Pediatrics, Pharmacology and Physiology, George Washington University School of Medicine, Washington DC 20037, USA

## Abstract

**Background:**

Nucleotide-actived P2Y receptors play critical roles in the growth of tumor cells by regulating cellular proliferation, differentiation and survival.

**Results:**

Here we demonstrate that an avian P2Y purinoceptor (tP2YR) with unique pharmacological and signal transduction properties induces morphologic and growth transformation of rodent fibroblasts. tP2YR induced a transformed phenotype similar to the *mas *oncogene, a G protein-coupled receptor which causes transformation by activation of Rac-dependent pathways. tP2YR-transformed cells exhibited increased steady-state activation of Rac1 and RhoA. Like activated Rho GTPases, tP2YR cooperated with activated Raf and caused synergistic transformation of NIH3T3 cells. Our data indicate that the ability of tP2YR to cause transformation is due to its unique ability among purinergic receptors to simultaneously activate Gα_q _and Gα_i_. Co-expression of constitutively activated mutants of these two Gα subunits caused the same transformed phenotype as tP2YR and Mas. Furthermore, transformation by both tP2YR and Mas was blocked by pharmacological inhibition of Gα_I _by pertussis toxin (PTX) indicating an essential role for Gα_i _in transformation by these G-protein coupled receptors.

**Conclusions:**

Our data suggest that coordinated activation of Gα_q _and Gα_i _may link the tP2YR and possibility the Mas oncogene with signaling pathways resulting in activation of Rho family proteins to promote cellular transformation.

## Background

Extracellular nucleotides such as adenosine 5'-triphosphate (ATP), adenosine 5'-diphosphate (ADP), uridine 5'-diphosphate (UDP) and uridine 5'-triphosphate (UTP) interact with purinergic receptors to modulate a broad spectrum of physiological responses [1]. Purinoceptors can be divided based on their pharmacological profiles into two major types: P1- and P2-purinoceptors [2]. Additionally, P2 receptors can be further subdivided into P2X-purinergic receptors which form ligand gated ion channels and P2Y-purinergic receptors which couple to heterotrimeric G-proteins. Most commonly, P2Y receptors activate phospholipase C (PLC) but some also inhibit or stimulate adenylyl cyclase or modulate ion channel activity [3].

While the majority of studies indicate that activation of purinergic receptors inhibits tumor growth, some recent data suggest cell context-dependent differences where in some instances purinergic receptors may contribute to tumorigenesis. For example, multiple P2 receptor subtypes have been identified in a variety of transformed cell lines and human tumors and activation of these receptors regulates apoptosis, proliferation and differentiation [4]. Consequently, depending on the combination of purinoceptors expressed in a tumor cell line and the second messenger pathways stimulated upon activation, addition or release of ATP could potentially promote or inhibit tumor growth [4-6]. For instance, expression of P2Y_1 _receptors can inhibit and P2Y_2 _receptor stimulate proliferation of a melanoma cell line in response to ATP [7]. In addition to its growth promoting activity in melanomas, stimulation of P2Y_2 _receptors can stimulate cell proliferation in lung, breast, ovarian and endometrial cell lines [8-11]. In addition to a role in the promotion of cellular proliferation, a mutated P2Y_2 _receptor has been isolated in an expression screen to identify potential transforming genes expressed in a colorectal cancer cell line [12]. The oncogenic potential of this mutated P2Y_2 _receptor was confirmed in focus formation, soft agar and tumorigenicity assays in nude mice.

P2Y-purinoceptors can regulate a complex network of signaling pathways that may contribute to transformation. P2Y receptors can couple to Gα_q _or Gα_i _stimulating PLC either by activation of Gα_q _or release of Gβγ following Gα_i _activation [13]. Furthermore, increases in the activity of multiple kinases, which can promote proliferation have been reported following stimulation of P2Y receptors. These include Pyk2, protein kinase C (PKC), and the ERK, p38 and JNK mitogen-activated protein kinase cascades [13]. In addition, an RGD domain in P2Y_2 _links this receptor to activation of G_12/13 _and G_o _stimulating RhoGEF/Rho and RacGEF/Rac1, respectively [14,15]. While numerous second messenger pathways can be activated by P2Y receptors, the pathways that contribute to the promotion of a transformed phenotype remain unexplored.

Other G-protein coupled receptors (GPCR) oncogenes cause tumorigenic transformation of NIH3T3 cells by activating Rho family proteins [16]. Rho proteins are members of the Ras superfamily of small GTPases that function as GDP/GTP-regulated molecular switches to modulate a variety of cellular processes including actin cytoskeletal organization, gene transcription and cell cycle progression [17,18]. Specific Rho family proteins regulate the reorganization of distinct actin cytoskeletal structures. Cdc42 stimulates the formation of filopodia, Rac1 formation of lamellipodia and membrane ruffling and RhoA formation of actin stress fibers and focal adhesions [19]. Rho family proteins themselves can cause tumorigenic transformation of rodent fibroblasts and mediate transformation by a number of oncogenes including Ras and Dbl [20]. A number of studies indicate that transformation by GPCRs is also dependent on Rho family proteins [16]. For example, Mas transformation is dependent on Rac1 function [21], whereas, G2A, Par-1, and M1 acetylcholine receptor transformation is mediated in part by activation of RhoA or a RhoA-related protein [22-24].

We cloned an avian P2Y purinergic receptor (tP2YR) which shows strongest amino acid sequence identity with the mammalian UTP-selective P2Y_4 _receptor [25]. tP2YR has a unique pharmacological profile [26]. It is preferentially activated by nucleoside triphosphates and exhibits low affinity for nucleoside diphosphates. However, it displays no selectivity for the nucleotide base moiety being activated by UTP, UTP, GTP, CTP, ITP and XTP. Furthermore, in addition to stimulating PLC through the Gα_q/11 _class of heterotrimeric G-proteins, activation of tP2YR also results in pertussis toxin-sensitive inhibition of adenylyl cyclase indicating that this receptor also has the unique ability among purinoceptors to simultaneously couple to Gα_q _and Gα_i _[25,26]. During the course of our characterization of tP2YR, we observed that this receptor induced striking morphologic transformation of 1321N1 astrocytoma cells prompting us to determine whether the tP2YR could cause morphologic and growth transformation of NIH3T3 cells. We found that tP2YR caused activation of Rho family proteins and requires the simultaneous activation of both Gα_q _and Gα_I _for transformation. We propose that this purinergic receptor stimulates cell growth and transformation through the coordinated activation of both Gα_q _and Gα_i_-dependent signaling pathways resulting in activation of Rho family proteins.

## Results

### Expression of the tP2YR induces altered cell morphology in 132N1 astrocytoma cells

**Figure 1:**
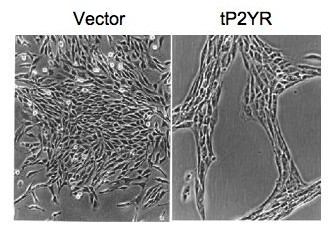
**Expression of tP2YR in 1321N1 astrocytoma cells results in an altered cell morphology**. 1321N1 cells were stably infected with expression constructs encoding either tP2YR or empty retrovirus vector as indicated (1000× magnification).

To study the pharmacological selectivity of tP2YR, we generated a number of clonal 1321N1 human astrocytoma cell lines stably infected with a retrovirus expression vector encoding the tP2YR. Surprisingly, a number of these cell lines exhibited an unusual morphologic and growth alteration not observed with the expression of other purinergic receptors in 1321N1 cells (data not shown). While vector-infected cells were unchanged in appearance from control untransfected 1321N1 cells and formed confluent monolayers of cells, the tP2YR-expressing cells displayed an elongated morphology and showed string-like patterns and cell clusters (Figure 1). Furthermore, the analyses of multiple, independent clonal cell lines indicated that this altered morphology correlated with elevated basal phospholipase C activity (data not shown). This ability to cause morphological transformation of the 1321N1 astrocytoma cell line was unique among the numerous receptors that we have evaluated previously, including the mammalian P2Y_2 _receptor (Boyer and Harden, unpublished observation).

### Expression of tP2YR causes transformation of NIH3T3 cells

**Figure 2:**
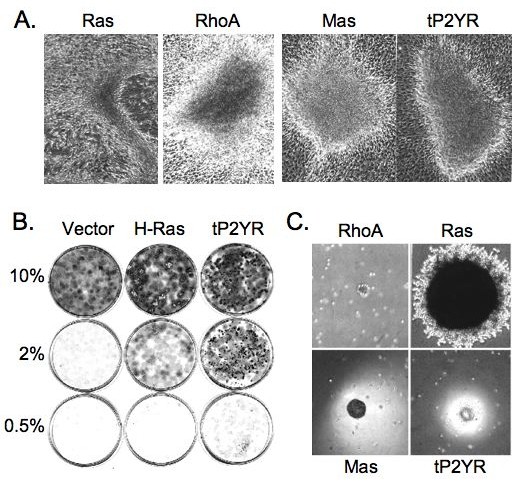
**Expression of tP2YR causes tumorigenic transformation of NIH3T3 cells**. **A**. Expression of tP2YR in NIH3T3 cells induces the formation of transformed foci. NIH3T3 cells were transfected with empty vector, H-Ras(61L) or RhoA(63L), the Mas Oncogene or tP2YR. The appearance of foci of transformed cells was evaluated 14 to 16 days after transfection (40× magnification). **B**. tP2YR-expressing NIH3T3 cells exhibit serum independent growth. NIH3T3 cells stably-transfected with the empty vector or encoding activated H-Ras(61L) or tP2YR were plated at a density of 10^3 ^cells per 60 mm dish in growth medium supplemented with 10% calf serum. After 24 hr, the growth medium was changed to media supplemented with 0.5%, 2% or 10% calf serum. The cultures were then maintained for 2 weeks and stained with 4% crystal violet to better visualize the cells. **C**. tP2YR-expressing NIH3T3 cells show anchorage-independent growth. NIH3T3 cells stably-transfected with pZIP-NeoSV(x)1 retrovirus vectors encoding the indicated proteins were plated in soft agar at a density of 10^3 ^[for Ras(61L)] or 10^4 ^[for RhoA(63L), Mas and tP2YR)] cells per 60 mm dish. The appearance of colonies of proliferating cells was evaluated after 3 weeks (40× magnification).

The altered morphology of tP2YR-expressing 1321N1 cells suggested that this GPCR may exhibit transforming properties. To investigate this hypothesis further, we determined if the tP2YR could also cause transformation NIH3T3 cells, a nontransformed immortalized mouse fibroblast cell line that we and others have shown to be sensitive to morphologic and growth transformation by Rho GTPase activation [27]. This experimental paradigm has provided a sensitive assay to detect the transforming activity of other GPCR oncoproteins and has been instrumental in dissecting the signal transduction pathways responsible for their transforming activity [21-24]. Using a focus formation assay, we found that transfection of pZIP-tP2YR in to NIH3T3 cells caused the appearance of foci of transformed cells (Figure 2A). Interestingly, the appearance of these foci was distinct from the foci caused by activated Ras(61L), which are comprised of highly refractile, elongated cells and instead were similar to those caused by activated RhoA(63L), which are comprised of densely-packed, nonrefratile cells. Furthermore, tP2YR-induced foci were similar in appearance to those induced by two other transforming GPCRs that cause transformation by activation of either Rac1-dependent (Mas) or RhoA-dependent (G2A) signaling pathways [21,22].

To determine if overexpression of the tP2YR reduced the requirement of NIH3T3 cells for serum growth factors, tP2YR-expressing cells were plated at low density in growth medium supplemented with 10% calf serum. After 24 hours, the cultures were switched to growth media supplemented with 10%, 2% or 0.5% calf serum. Untransformed NIH3T3 cells require growth medium supplemented with 10% calf serum for efficient growth in culture. As expected, cells transformed by activated Ras continue to proliferate in 2% or 0.5% serum, whereas vector-transfected control cells exhibited very limited proliferation in growth medium supplemented with 2% or 0.5% serum (Figure 2B). Similar to activated Ras-expressing cells, tP2YR-expressing cells formed dense colonies even in 0.5% serum, indicating that expression of the tP2YR can reduce the growth requirement of NIH3T3 cells for serum growth factors.

To further characterize the ability of tP2YR to cause growth transformation of NIH3T3 cells, we assessed the ability of tP2YR-expressing cells to exhibit anchorage- and serum-independent growth. For these experiments, we isolated NIH3T3 cells stably transfected with expression constructs encoding either H-Ras(61L), RhoA(63L), Mas, tP2YR, or the pZIP-NeoSV(x)1 empty vector control. Following selection in growth medium supplemented with G418, multiple antibiotic-resistant colonies (>100) were pooled together to establish stable cell lines to be used for these analyses. To assay the ability of the tP2YR expressing cells to exhibit anchorage-independent growth, each pooled cell line was suspended in soft agar. As described previously, cells expressing activated Ras or RhoA formed colonies in soft agar [28] and Figure 2C). The tP2YR-expressing cells also readily formed colonies in soft agar indicating that expression of tP2YR can cause anchorage-independent growth. Interestingly, tP2YR-expressing colonies were similar in size and morphology to those formed by Mas-transformed cells. For example, these colonies did not spread laterally in the agar as mutant Ras-expressing colonies, but instead formed clusters of cells that protruded out of the plane of the agar.

### Similar to activators of Rho family proteins, tP2YR cooperates with activated Raf1 in focus formation assays

**Figure 3:**
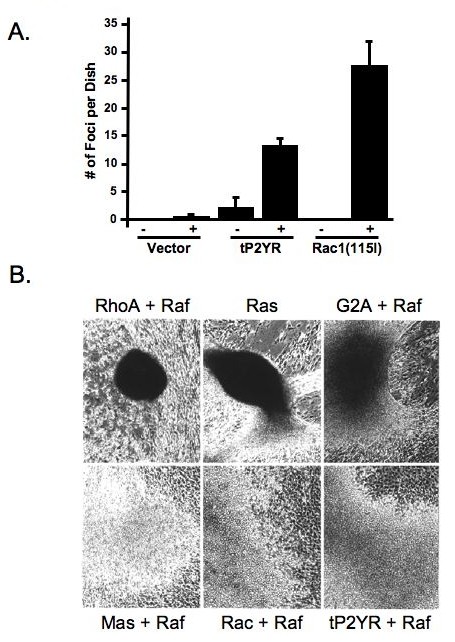
**tP2yR synergizes with activated Raf1 to induce foci with similar characteristics to activated Rac1**. **A**. Quantitation of the number of foci induced when tP2YR or activated Rac1(115I) were co-transfected with activated Raf-1(340D). NIH3T3 cells were co-transfected with 1 μg of pZIP-NeoSV(x)1 retrovirus expression plasmids encoding tP2YR or Rac1(115I) and either 1 μg of the empty pZIP-NeoSV(x)1 plasmid DNA (-) or encoding the weakly activated Raf-1(340D) mutant (+). The appearance of foci of transformed cells was quantitated after 14 days. Data shown are the mean ± standard error for triplicate plates and are representative of three independent assays. **B**. The appearance of transformed foci induced in NIH3T3 cells were co-transfected with activated Raf-1(340D) is similar to those induced by activated Rac(115I) and distinct from those induced by activated Rho(63L). pZIP-NeoSV(x)1 expression vectors encoding RhoA(63L), G2A, Mas, Rac1(115I) or tP2YR were co-transfected with pZIP-*raf*-1(340D). pZIP-H-*ras*(61L) was co-transfected with the empty pZIP-NeoSV(x)1 plasmid DNA. The appearance of foci of transformed cells was monitored 14 days after transfection (40× magnification).

The morphological appearance of the tP2YR-induced foci suggested that tP2YR might cause transformation by activation of Rho family proteins. One well-characterized property of transformation by Rho proteins and their activators is their ability to cooperate with activated Raf-1 to cause synergistic transformation of NIH3T3 cells [28-32]. To determine if tP2YR could also cooperate with activated Raf-1, plasmids encoding activated Raf-1(340D) were co-transfected at concentrations that resulted in very low, or no, focus-formation along with either tP2YR, activated Rac1(115I) as a positive control, or the empty vector negative control. As we observed previously, co-expression of activated Rac1(115I) with Raf-1(340D) caused a 100-fold enhancement of focus-forming activity over that seen with expression of Rac1(115I) or Raf alone [28]. Similarly, as shown in Figure 3A, co-transfection of tP2YR with activated Raf caused a 15- to 20-fold synergistic enhancement of focus-forming activity. These results indicate that like activators of Rho proteins, tP2YR can cooperate in a focus formation assay with activated Raf-1.

In addition to increasing the number of tP2YR-induced foci, co-expression with activated Raf altered the morphology of the transformed foci (Figure 3B). As described previously, co-expression of activated Raf-1 with either RhoA or G2A causes the appearance of transformed foci that are similar to those caused by activated Ras and contain elongated, highly refractile cells [21]. In contrast, co-transfection of activated Rac1 or Mas with activated Raf-1 caused the appearance of foci that were distinct, and contained rounded cells with a non-refractile appearance [21]. The transformed foci caused by co-expression of Raf-1 and tP2YR were indistinguishable in appearance from those caused by expression of activated Raf-1 and either activated Rac1 or Mas, suggesting that tP2YR may activate Rac1.

### tP2YR causes activation of both Rac1 and RhoA

**Figure 4:**
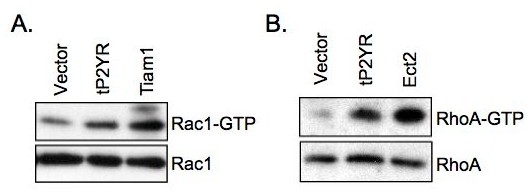
**Expression of tP2YR results in lamellipodia formation and activation of both Rac1 and RhoA**. NIH3T3 cells were transiently transfected with either empty pcDNA3 expression vector or those encoding tP2YR, Tiam1-C1199 (**A**, potent activator of Rac1) or Ect2-DH/PH/C (**B**, potent activator of RhoA). Activated GTP-bound Rac1 and RhoA were isolated in pull-down assays with GST-Pak-PBD and GST-Rhotekin-RBD, respectively.

The appearance of tP2YR foci suggests that it causes transformation by activation of Rac1 or a related protein. To address this possibility, we assessed the ability of tP2YR to activate Rac1 and RhoA in pull-down assays. These assays take advantage of the increased affinity of activated Rac1 or RhoA for the isolated Rac/Rho binding domains (RBDs) of Pak and Rhotekin, effectors of Rac and RhoA, respectively. For these experiments, NIH3T3 cells were transfected with either pcDNA3 or tP2YR along with either Tiam1-C1199 (positive control for Rac1 activation) or Ect2-DH/PH/C (positive control for RhoA activation). Cell lysates were incubated with GST-Pak-RBD to assess Rac1 activation or GST-Rhotekin-RBD to assess RhoA activation. As shown in Figure 4B and 4C, expression of tP2YR increased activation of both Rac1 and RhoA. These data indicate that tP2YR can cause activation of Rho family proteins.

### The tP2YR causes transformation by coordinated activation of Gα_i _and Gα_q_

**Figure 5:**
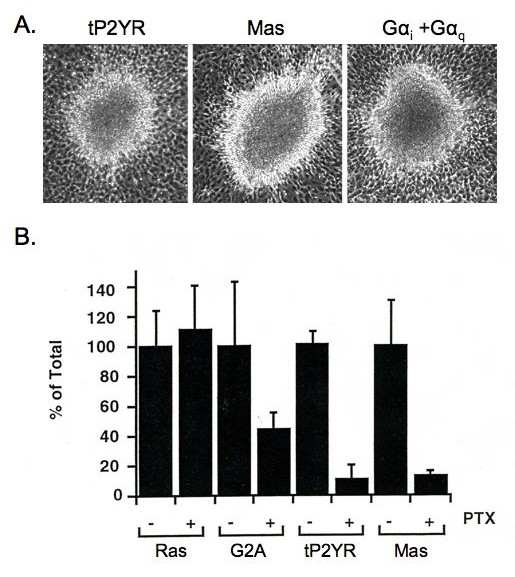
**Transformation of NIH3T3 cells by tP2YR is caused by coordinated activation of Gα_i _and Gα_q_**. **A**. Gα_i _and Gα_q _cooperate and cause the appearance of transformed foci similar to those caused by tP2YR. NIH3T3 cells were transfected with pZIP-NeoSV(x)1 expression vectors encoding tP2YR or Mas, or co-transfected with vectors encoding constitutively activated mutants of Gα_I2_(QL) and Gα_q_(QL). The appearance of foci of transformed cells was monitored 3 weeks after transfection (40× magnification). **B.** Transformation by tP2YR and Mas requires Gα_i _protein function. NIH3T3 cells were transfected with pZIP-NeoSV(X)1 expression vectors encoding H-Ras(61L) (25 ng per 60 mm dish), G2A (2 μg per dish), tP2YR (2 μg per dish) or Mas (1 μg per dish). Cultures were then maintained in growth medium (-) or medium supplemented with 100 ng per ml PTX (+) for 14 to 16 days after which the number of foci were quantitated. Data shown are expressed as the mean of the percent of the total number of foci in the untreated dishes ± standard error and are representative of two separate assays performed in triplicate.

We next investigated which Gα subunit(s) may link tP2YR to Rho protein activation. P2Y-purinergic receptors can couple to either Gα_q_, activating PLC or to Gα_i _inhibiting adenylyl cyclase activity. Typically purinergic receptors do not couple to both of these G-proteins. However, our previous studies indicated that this tP2YR has the unique ability to robustly activate both Gα_q _and Gα_i _[25,26]. Therefore, we sought to determine if activation of either or both of these Gα subunits may contribute to tP2YR transforming activity by examining the ability of constitutively activated mutants of Gα_i _and Gα_q _either, alone, or in combination, to cause focus-formation in NIH3T3 cells. When transfected alone, neither activated Gα_i_, nor activated Gα_q _caused the appearance of transformed foci in our NIH3T3 cells (data not shown). However, co-expression of activated Gα_i _and Gα_q _caused the appearance of transformed foci that were identical in appearance to those induced by the tP2YR or Mas (Figure 5A). These results suggested that the coordinate activation of both Gα_i _and Gα_q _by the tP2YR maybe responsible for its transforming activity.

To examine the requirement for Gα_i _activation for focus formation by these GPCR oncogenes, NIH3T3 cells were transfected with expression constructs encoding either Mas, tP2YR, G2A, or Ras(61L) and the transfected cultures were maintained in the presence or absence of PTX to inactivate Gα_i_. Treatment of cells with PTX caused a 90% inhibition of Mas- and tP2YR-induced focus-formation (Figure 5B). In contrast, PTX treatment did not cause any reduction in oncogenic Ras-induced focus formation, indicating that the reduction seen with tP2YR was not due to nonspecific inhibition by PTX. Interestingly, PTX also caused a 60% reduction in G2A-induced focus-formation. We previously showed that G2A causes transformation by activation of Gα13, which in turn causes activation of RhoA by stimulation of the Lsc/p115 RhoGEF [22,23]. These data suggest that GPCR oncogenes such as Mas, tP2YR and to a lesser extent G2A rely on Gα_i _activity for induction of a transformed phenotype.

## Discussion

Purinergic receptors regulate a number of physiological functions and recent data indicate that purinergic signaling may also play an important role in both the promotion and inhibition of tumor growth [4,5]. Here we present evidence that an avian P2Y purinoceptor (tP2YR) with unique pharmacological and signal transduction properties induces morphologic and growth transformation. tP2YR expressing NIH3T3 cells exhibited loss of density-dependent inhibition of growth, proliferated in the absence of attachment to the substratum, and displayed a reduced requirement for serum growth factors. Furthermore, our analyses suggest that the tP2YR causes transformation by activation of Rho proteins. The foci caused by tP2YR expression are identical in appearance to those caused by another transforming GPCR, Mas that causes transformation by activation of Rac-dependent signaling pathways [21]. Furthermore, tP2YR causes activation of both Rac1 and RhoA in an *in vitro *biochemical assay. Finally, we present data suggesting that tP2YR, as well as Mas, cause transformation by coordinate activation of Gα_i _and Gα_q_.

### tP2YR causes transformation by activation of Rho proteins

While our *in vitro *Rac1 and RhoA activation assays indicate that tP2YR can activate both Rho family proteins, based on the appearance of transformed foci we favor the interpretation that activation of Rac1 (or a related protein) maybe more important for the transformed phenotype induced by tP2YR. For example, the appearance of tP2YR-induced foci was identical to those induced by expression of the Mas oncogene, which causes transformation by activation of Rac1 rather than RhoA [21]. In contrast, G-proteins and GPCRs such as Gα_12/13 _and G2A cause the appearance of Rho-like foci and require RhoA protein function for transformation [22-24,33,34]. Additionally, tP2YR cooperated with activated Raf in a focus formation assay causing the appearance of foci that resemble those by activated Raf plus Rac1 or Rac1 activators such as Mas [21]. This is in contrast to the appearance of foci induced by activated Raf plus RhoA or RhoA activators such as G2A [22]. These data suggest that while tP2YR is capable of activating both Rac1 and RhoA in an *in vitro *biochemistry assay; the morphologically predominant phenotype induced by expression of tP2YR suggests that Rac1 activation maybe more important for the transforming activity of this oncogene.

### tP2YR causes transformation by the coordinated activation of both Gα_i _and Gα_q_

Treatment of cells with ATP can influence cell growth and proliferation in opposing ways depending on the cell type and the combination of purinoceptors expressed. In rare instances, stimulation of P2Y receptors can promote cellular proliferation and oncogenic transformation [5,6,12,13]. Since the ability of tP2YR to cause tumorigenic transformation is unique among the receptors that we have characterized, the possibility remains that this avian receptor is constitutively activated. One characteristic of the tP2YR that sets it apart from other purinoceptors is its ability to simultaneously activate both Gα_i _and Gα_q _[25,26]. This observation prompted us to test the hypothesis that transformation by tP2YR maybe due to the coordinated activation of both Gα_i _and Gα_q_. We found that while neither Gα_i _nor Gα_q _when expressed alone in NIH3T3 cells induced focus formation, when co-expressed, these proteins induced the appearance of transformed foci. Furthermore, the morphology of Gα_i _and Gα_q _induced foci was identical to those induced by expression of either tP2YR or Mas indicating that similar pathways may be activated to promote transformation. While previous studies have demonstrated only a mild focus formation activity of Gα_q _in NIH3T3 cells [35], others indicate that Gα_q _expression induces apoptotic cell death [36,37], we have been able to establish stable cell lines that express Gα_q _using a relatively weak cytomegalovirus promoter (pcDNA3). Furthermore, focus formation was not observed in these stable cell lines indicating that Gα_q _expression alone is not transforming in the NIH3T3 strain used in these studies. Nor was Gα_q _transforming when co-expressed with activated Raf (data not shown). Finally, focus formation by both Mas and tP2YR requires Gα_i _activity supporting the hypothesis that it is the coordinated activation of these two Gα subunits, which leads to growth transformation by these GPCR oncoproteins.

How coordinated activation of Gα_i _and Gα_q _by tP2YR leads to activation of Rho proteins remains unknown. The mammalian P2Y_2 _receptor can activate both RhoA and Rac1 through interaction with integrins mediated by an RGD sequence motif located in the first extracellular loop of the receptor [13]. The interaction of the P2Y_2 _receptor with integrins is necessary for coupling to Gα_o _leading to activation of Rac1 and Gα_12 _and to activation of RhoA but is not required for activation of Gα_q _[14]. Since, the tP2YR does not contain an RGD sequence, it is likely that activation of Rho proteins are mediated by this alternative pathway. Previous studies have found that the activation of Gα_i _and Gα_q _by expression of multiple P2Y receptors on the same cell can cause a synergistic rise in second messenger production [13]. It is possible that this synergistic activation of second messengers can lead to Rho activation.

## Conclusions

Our data suggest that overexpression of the tP2YR causes growth transformation of NIH3T3 cells via a coordinate activation of Gα_i _and Gα_q_, leading to activation of Rho proteins. These studies provide novel insight into the mechanism by which GPCR signaling can promote tumorigenesis.

## Methods

### Molecular constructs

The tP2YR mammalian retrovirus expression construct pLXSN-*tP2YR *has been described previously [25]. pZIP-NeoSV(x)1 retrovirus expression vectors encoding wild type Mas, and the constitutively activated and transforming Rac1(61L), Rac1(115I), H-Ras(61L), RhoA(61L), or Raf-1(340D) mutant proteins and pCDNA3 expression vectors encoding GTPase-deficient, constitutively activated mutants of Gα_q_(QL), Gα_i_(QL), Rac1(12V), and RhoA(12V) have also been described previously [38,39].

### Cell culture and transformation assays

1321N1 human astrocytoma cells were grown in Dulbecco's minimum Eagle's medium (DMEM) supplemented with 5% fetal calf serum. Infection of 1321N1 astrocytoma cells was done as described [25]. NIH3T3 mouse fibroblasts were grown in DMEM supplemented with 10% calf serum and transfected as described previously [40]. For focus formation assays, 60 mm dishes were transfected and maintained in growth medium for three weeks. Individual foci were either photographed and/or stained with 0.4% crystal violet before quantitation. To evaluate the ability of pertussis toxin (PTX) treatment to inhibit transformation, focus formation assays were performed as above in growth medium supplemented with 100 ng/ml of PTX (Sigma). Growth medium and PTX were replaced every other day for 14-16 days. For generation of stable cell lines, transfected cultures were maintained in growth medium supplemented with 400 μg/ml G418 (Geneticin; GIBCO-BRL) and following selection, multiple G418-resistant colonies (>100) were pooled. Assays to examine growth in soft agar and low serum have been described previously [41].

### Rac1 and RhoA activation assays

Assays to determine cellular Rac1-GTP and RhoA-GTP levels were performed as described previously [42,43]. Briefly, NIH3T3 cells were transiently transfected with either empty pcDNA3 expression vector or those encoding tP2YR, the C-terminal 1199 amino acids of Tiam-1, C1199 [44] or the DH/PH/C domain of Ect2 [45], using Lipofectamine Plus Reagent (Invitrogen Inc.). Cells were grown for 24 h in DMEM supplemented with 0.5% fetal calf serum. Cell lysates were incubated with GTP-dependent binding domains for Pak1 (GST-PAK-RBD) or Rhotekin (GST-Rhotekin-RBD) as we have described previously [42,43].

## Abbreviations

tP2YR: avian P2Y purinoceptor; GPCR: G-protein coupled receptor; PTX: pertussis toxin; ATP: adenosine 5'-triphosphate; ADP: adenosine 5'-diphosphate; UDP: uridine 5'-diphosphate; UTP: uridine 5'-triphosphate; GDP: guanosine diphosphate; GTP: guanosine triphosphate; PLC: phospholipase C; PKC: protein kinase C;

## Competing interests

JLB is currently an employee of Inspire Pharmaceuticals Inc Durham NC. With this exception, the authors declare that they have no other competing interests.

## Authors' contributions

AS, Carried out Rac1 and RhoA activation assays and helped to draft the manuscript. JLB made the initial observation that tP2yR caused a transformed phenotype, guided these studies and helped to draft the manuscript. CJD guided these studies and helped to draft the manuscript. CJD guided these studies and helped to draft the manuscript. IEZ performed all other experiments and drafted the manuscript.
